# CIS6/413: Design of a Computer-Based Patient Record System (ARISTOPHANES) for Medical Departments: Implementation for Surgery Wards

**DOI:** 10.2196/jmir.1.suppl1.e11

**Published:** 1999-09-19

**Authors:** A Lazakidou, J Braun, T Tolxdorff

**Affiliations:** ^1^Free University of BerlinBerlinGermany

**Keywords:** Information Systems, Databases, Electronic Patient Records

## Abstract

**Introduction:**

Today, the demand for computer-based patient records to improve the quality of patient care and to reduce costs in health services is generally recognized. The new electronic patient record system (ARISTOPHANES) is based on a self-developed relational data model with a star-shaped topology. An hierarchical structure has been chosen for the user interface. ARISTOPHANES has been developed for use in clinical workstations taking into account varied requirements, user acceptance, and future extension.

**Methods:**

For the design of the electronic patient records was used the software "Oracle Forms 5.0". An Oracle database has been generated and loaded with test data. The designed forms are currently being implemented using "SQL*Plus 8.0" by Oracle.

**Results:**

We used a self-developed star-shaped relational data model, which is highly flexible with regard to the modification and extension of the data structure. The design of the electronic patient records has taken into account the varied demands of the medical partners in order to guarantee user acceptance. The hierarchical topology that has been chosen renders the designed electronic patient record system flexible and efficient, such that further departments and regulatory changes can be integrated within a short time. General functions required by all departments are contained in the main menu. These include patient data, case details, medical documentation, care documentation, personnel details, and business partners. Specific functions are included at lower levels for the demands of the surgery departments, such as anaesthesia record, operation record, present status record.

**Discussion:**

The complexity of user acceptance is reflected by (i) the different groups of users of Electronic Healthcare Record systems (physicians, nurses, managers, researchers) together with their various requirements, and (ii) the different contexts of use (e.g., support in normal or emergency situations, access for research, and mobile access). Commercial providers have developed electronic patient record systems, that often turn out to be too inflexible for the requirements of a university setting and need to be customized considerably. To ensure user acceptance, we analyzed the demands together with clinical partners from the surgery departments. It is expected that the ARISTOPHANES system will also be attractive to other medical departments in light of the overall advantages such as paperless documentation, reliable patient data transfer, improved settlement of accounts, and easing the burden on care personnel.

